# Long-term trends in grassland bird relative abundance on focal grassland landscapes in Missouri

**DOI:** 10.1371/journal.pone.0281965

**Published:** 2023-03-09

**Authors:** Alexander R. Schindler, Hadley I. A. Boehm, Tyler F. Beckerman, Thomas W. Bonnot, Frances M. DiDonato, Alisha R. Mosloff, Mitch D. Weegman, Sarah W. Kendrick

**Affiliations:** 1 Department of Biology, University of Saskatchewan, Saskatoon, SK, Canada; 2 School of Natural Resources, University of Missouri, Columbia, MO, United States of America; 3 Missouri Cooperative Fish and Wildlife Research Unit, Columbia, MO, United States of America; 4 Missouri Department of Conservation, Jefferson City, MO, United States of America; Sichuan University, CHINA

## Abstract

North American grassland birds have widely declined over the past 50 years, largely due to anthropogenic-driven loss of native prairie habitat. In response to these declines, many conservation programs have been implemented to help secure wildlife habitat on private and public lands. The Grasslands Coalition is one such initiative established to advance the conservation of grassland birds in Missouri. The Missouri Department of Conservation conducted annual point count surveys for comparison of grassland bird relative abundance between focal grassland areas and nearby paired (i.e., containing no targeted management) sites. We analyzed 17 years of point count data with a generalized linear mixed model in a Bayesian framework to estimate relative abundance and trends across focal or paired sites for nine bird species of management interest that rely on grasslands: barn swallow (*Hirundo rustica*), brown-headed cowbird (*Molothrus ater*), dickcissel (*Spiza americana*), eastern meadowlark (*Sturnella magna*), grasshopper sparrow (*Ammodramus savannarum*), Henslow’s sparrow (*A*. *henslowii*), horned lark (*Eremophila alpestris*), northern bobwhite (*Colinus virginianus*), and red-winged blackbird (*Agelaius phoeniceus*). Relative abundance of all species except eastern meadowlarks declined regionally. Relative abundance of barn swallows, brown-headed cowbirds, dickcissels, eastern meadowlarks, Henslow’s sparrows, and northern bobwhites was higher in focal than paired sites, though relative abundance trends were only improved in focal vs. paired areas for dickcissels and Henslow’s sparrows. Relative abundance increased with increasing grassland cover at the local (250-m radius) scale for all species except horned larks and red-winged blackbirds and at the landscape (2,500-m radius) scale for all species except dickcissels, eastern meadowlarks, and northern bobwhites. Our results suggest focal areas contained greater relative abundances of several grassland species of concern, likely due to increased availability of grassland habitat at local and landscape scales. Further efforts to decrease landscape-scale fragmentation and improve habitat quality may be needed to achieve conservation goals.

## Introduction

North American birds have declined in abundance by nearly 3 billion individuals since 1970 [1]. Grassland bird species have declined at one of the fastest rates [1–3], with abundance declining by >53% from 1970–2017 [1]. Anthropogenic-driven habitat loss, including large-scale conversion of native prairie to agricultural crops, is a major cause of grassland bird declines [2]. More than 80% of grassland habitat (>97% of tallgrass prairie) has been lost in North America since the mid-1880s [4–6]. Most remaining prairie is managed for cattle production or left idle for wildlife habitat [7], and factors such as pesticide use, intensive grazing or haying, invasive plants, inadequate burning, and energy development have further degraded and fragmented remaining grasslands [3, 6, 8, 9].

There is a large body of literature demonstrating that both local and landscape-scale habitat features drive grassland bird abundance [10–12]. It is therefore important to consider multiple spatial scales when assessing the success of management programs, as different landscape features could be driving changes in abundance at different spatial scales [13, 14]. Multi-scale habitat assessments are particularly important to optimize conservation outcomes among species, as life history differences can result in varying responses to landscape features, influencing abundance and community composition across scales [15–17].

Tallgrass prairie covered about one-third of Missouri prior to European settlement, yet less than a half of one percent of the original cover remains today [18, 19]. In response to the dramatic decline in tallgrass prairie, the Missouri Prairie Foundation and the Missouri Department of Conservation established the Grasslands Coalition (GC) in 1998 to help preserve the state’s remaining tallgrass prairie, educate the public on the importance of grasslands, and improve grassland habitat in areas that can make a significant and lasting difference to grassland bird species [20]. This includes promoting management regimes that conserve and enhance the natural ecosystem functions of both remnant and restored prairies [21, 22]. GC partners conducted annual roadside point count surveys for grassland bird species to compare relative abundances in focal (i.e., areas with targeted grassland habitat management as part of the GC) and paired (i.e., areas the GC did not target for habitat management) sites. Such monitoring efforts are an important part of conservation programs, as they allow managers to formally assess if conservation goals are being achieved.

In this study, we used a generalized linear mixed model in a Bayesian framework [23] to assess the effectiveness of GC actions on trends in annual relative abundance of nine grassland bird species of management interest. A common objective of grassland management is to increase the amount of grassland across multiple spatial scales; therefore, we also evaluated the effects of grassland cover at local and landscape scales on grassland bird relative abundance. Our objectives were to 1) determine trends in relative abundance for grassland bird species of interest on grassland focal sites and paired sites in Missouri, 2) determine differences in grassland bird relative abundance and relative abundance trends between focal and paired grassland sites, and 3) quantify the effect of percent grassland cover at local and landscape scales on bird relative abundance.

## Methods

### Study area

**Fig 1:**
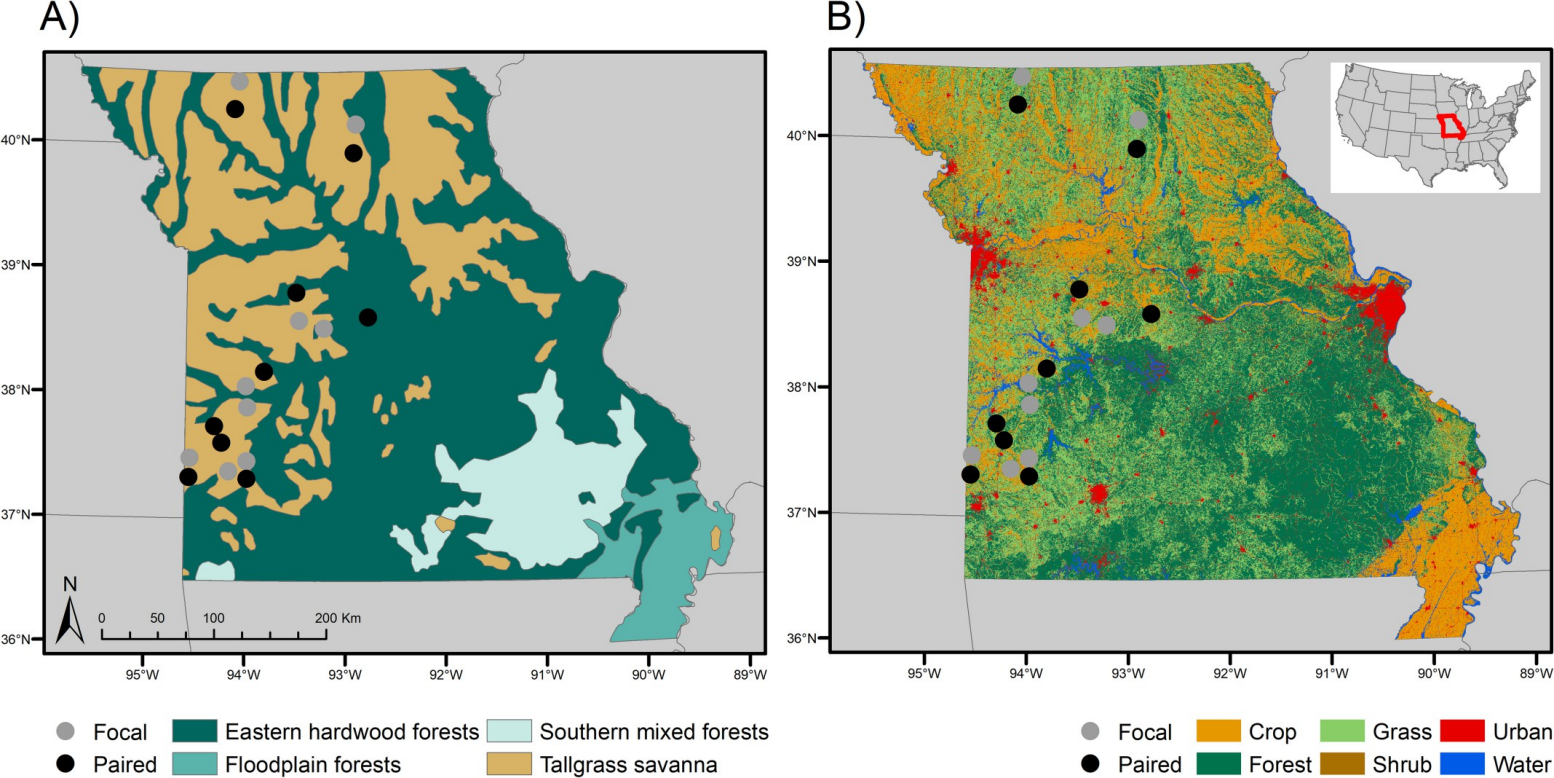
Map of study areas. Locations of nine focal and paired study areas in Missouri across A) potential natural vegetation (created by ARS with data from [25]) and B) land cover (created by ARS with data from [26]).

GC partners identified nine areas (hereafter “focal” areas, Fig 1) in the tallgrass prairie region in western and northwestern Missouri. Focal areas ranged from 4,230 to 18,952 ha and were identified based on a combination of land cover characteristics and locations of grassland bird communities to prioritize areas with greatest potential for effective grassland management [21, 22]. Each focal grassland area attempted to contain an ~800-ha core of continuous grassland surrounded by a ~3,200-ha buffer or matrix that contained an additional ~800 ha of grassland tracts of at least 40 ha in area based on the Partners in Flight Bird Conservation Area model [24]. Management actions within focal areas included removal of trees and shrubs, native plant restoration, prescribed fire, rotational grazing, and/or control of exotic and invasive species, and were carried out through a combination of incentive-based programs with private landowners, public land management, and grant-funded restoration of public and private lands [21, 22]. All focal areas did not adhere to the one single management plan due to variation in land ownership, available work teams, feasibility of management actions on large landscapes, etc. Rather, focal areas were those that often surrounded a tract of protected grassland habitat and received ongoing management actions to benefit and promote vegetative biodiversity and diversity of grassland structural, including prevention of succession to shrublands, disturbance to maintain a variety of grassland structures, and control of invasive plant species.

For each focal area, GC partners identified a nearby paired area (mean distance between focal and paired areas was 15.89 km) of similar vegetative composition, size, and landscape characteristics to compare with each focal area and assess the effectiveness of focal area designation. Paired areas generally contained grazed pastures or row crops without targeted efforts for native grassland maintenance or restoration. While management on focal areas was generally intended to be more intensive and directed at grassland species, specific management plans and detailed records of the actions listed above were lacking, thus precluding direct evaluation of effects of individual management actions.

### Bird surveys

Agency staff and trained volunteers conducted surveys near focal and paired areas to detect major differences in bird communities and relative abundance to test the Partners in Flight Bird Conservation Area Concept [24]. Survey routes comprised 50 roadside point counts at 0.8-km intervals along roads for each of the nine focal and paired areas (i.e., 18 total routes). Following North American Breeding Bird Survey protocol, observers listened for bird detections for three minutes at each point, recording the number of each species seen and/or heard before driving to the next point and repeating [27]. These surveys occurred once per route annually between 25 May and 5 July from 2001–2017 [21, 22]. Because many of the focal and paired areas contained large, contiguous grassland patches, roads used in point counts were often adjacent to rather than within these respective grassland areas.

### Study species

We analyzed survey count data for nine obligate or facultative grassland species, including barn swallow (*Hirundo rustica*), brown-headed cowbird (*Molothrus ater*), dickcissel (*Spiza americana*), eastern meadowlark (*Sturnella magna*), grasshopper sparrow (*Ammodramus savannarum*), Henslow’s sparrow (*A*. *henslowii*), horned lark (*Eremophila alpestris*), northern bobwhite (*Colinus virginianus*), and red-winged blackbird (*Agelaius phoeniceus*). These species represent a range of preferences for grassland composition (e.g., natural prairies and grassland-cropland mosaics) and vegetative structures (e.g., tall rank grass, patchy grass, and bare ground), life histories (resident or migratory), and statuses (e.g., abundant or species of conservation concern) in Missouri [28, 29; Table 1]. Additionally, the brown-headed cowbird is a species of interest to managers due to its brood parasitic behavior, which can negatively affect breeding success of a variety of songbird species [30].

**Table 1:** Nine focal obligate and facultative grassland bird species selected for analysis.

Common Name	Scientific Name	MO Concern Score	Habitat preferences	Resident or Migratory (MO)
Barn swallow	*Hirundo rustica*	0	open areas including suburban parks, croplands, over open water	migratory
Brown-headed cowbird	*Molothrus ater*	0	grasslands with low and scattered trees, woodland edges, brushy thickets, prairies, fields, pastures, residential areas	resident/migratory
Dickcissel	*Spiza americana*	16	variety of grasslands with dense cover and high proportion of forbs	migratory
Eastern meadowlark	*Sturnella magna*	17	prairies, grasslands, fallow fields, pastures, harvested cultivated fields	resident
Grasshopper sparrow	*Ammodramus savannarum*	15	open grasslands with regular disturbance	migratory
Henslow’s sparrow	*Ammodramus henslowii*	17	tall, dense grass with longer disturbance intervals	migratory
Horned lark	*Eremophila alpestris*	0	areas with bare ground or short, sparse vegetation including prairies and heavily grazed pastures	resident
Northern bobwhite	*Colinus virginianus*	16	shrub obligate, large landscapes of native grassland, savannah, open woodland, croplands interspersed with suitable herbaceous and woody cover	resident
Red-winged blackbird	*Agelaius phoeniceus*	0	wetlands, sedge meadows, alfalfa fields, fallow fields, croplands, pastures, grasslands	resident

Shown are species common and scientific names, Missouri Concern Score as listed in the Missouri Bird Conservation Plan [28], habitat preferences [29], and whether the species is migratory or resident in Missouri. Missouri Concern Scores reflect vulnerabilities of each species based on 5 factors: population trends in Missouri, threats to breeding in Missouri, relative density in Missouri, global population size, and global breeding distribution. Conservation practitioners assigned a score from 1 (least concern) to 5 (greatest concern) for each of these factors. The Missouri Concern Score is the sum of scores of these 5 factors (see [28] for full scoring methodology). A Missouri Concern Score of 0 indicates the species was not included in the Missouri Bird Conservation Plan. While other bird species were recorded during the survey period, those data were too sparse to include in this analysis.

### Land cover data

We obtained land cover data for our study areas from the Cropland Data Layer for 2006–2017 [26]. The Cropland Data Layer combines National Land Cover Database [31] classifications with information on specific crop types at a 30-m spatial resolution and is ground-truthed with overall accuracy ranging from 76.5% - 92.4% for agricultural and 78% - 86.4% for nonagricultural classes [26, 31]. We defined “grassland” as classes comprised of clover/wildflowers, switchgrass, grass/pasture, alfalfa, or other hay/non alfalfa in the Cropland Data Layer. We calculated the percent grassland cover at two scales around survey locations (local scale: 250-m buffer around survey points [~20 ha], landscape scale: 2,500-m buffer around survey routes [~21563 ha]). These scales represent local- and landscape-scale habitat features with respect to our focal species’ home ranges [29] and are similar to scales used in other multi-scale habitat analyses [32–34]. Because these data were not available for the period 2001–2005, we used linear regression to develop a predictive model of grassland cover at both scales around points from existing data and extrapolated values for years with missing data.

Among all years, focal area sites had a mean of 54.0% grassland cover at the local (250-m buffer) scale and a mean of 51.9% grassland cover at the landscape (2,500-m buffer) scale (95% of all stops/routes between 5.4% - 92.2% and 32.7% - 73.7%, respectively). Among all years, paired area sites had a mean of 50.1% grassland cover at the local scale and a mean of 50.0% grassland cover at the landscape scale (95% of all stops/routes between 3.6% - 92.5% and 29.4% - 72.0%, respectively). Percent grassland was generally similar among focal and paired site pairings (S1 Fig).

### Analysis

We developed generalized linear mixed models [23] in a Bayesian framework to compare temporal trends in relative abundance of focal species between GC focal areas and their paired areas and the range of grassland cover on them. Because count data contained a large proportion (>25%) of non-detections (0 counts), we assumed data were generated from a zero-inflated Poisson process such that

$$y_{k,a,t}∼Poisson[\lambda_{k,a,t}\times (1-z_{k,a,t})],$$ (1)

where *y*_*k*,*a*,*t*_, represented count data from surveys at route *k* and point *a* in year *t*, *λ*_*k*,*a*,*t*_ represented the expectation of count data *y*_*k*,*a*,*t*_, and *z*_*k*,*a*,*t*_ was a Bernoulli random variable with probability *ω*, where *ω* ~ *U*(0, 1). Dickcissel count data did not contain a large proportion (<25%) of non-detections; thus, we assumed data for this species were Poisson distributed (see S1 Appendix). To account for overdispersion in the red-winged blackbird count data, we specified count data as coming from a negative binomial distribution (see S1 Appendix). Point count survey protocols did not contain methods to account for imperfect detection (e.g., distance sampling, time-to-first detection, repeated counts, etc.), therefore we were unable to correct for imperfect detection in our models.

We constructed ecological process models to describe changes in expected relative abundance with respect to the area’s focal or paired status and land cover. We defined these models as

$$log(\lambda_{k,a,t})=\beta_{1}\times x_{k}^{focal}+\beta_{2}\times x_{k,a,t}^{grass250}+\beta_{3}\times x_{k,t}^{grass2500}+\varepsilon_{k,t}$$ (2)

where *β*_1_, *β*_2_, and *β*_3_ described the effects of whether a site was a focal area or not and the grassland cover surrounding points at the local (250-m) and landscape (2,500-m) scales. We included *ε*_*k*,*t*_ as a random effect for route and year to account for a lack of independence and additional variation among sites and among years. We assumed *ε*_*k*,*t*_ was normally distributed such that

$$\varepsilon_{k,t}∼N(\alpha_{k},\sigma^{2}),\;\alpha_{k}∼N(\mu ,\tau^{2})$$ (3)

and specified prior distributions as *β*_1–3_ ~ *N*(0, 1000), *μ* ~ *N*(0, 1000), *σ* ~ *U*(0, 10), and *τ* ~ *U*(0, 10).

We estimated species- and route-specific trends (i.e., direction of change) in annual relative abundance (*R*) in the same model by taking the proportional change in relative abundance for subsequent years with the equation

$$R_{k,t-1}=(\lambda_{k,t}/\lambda_{k,t-1})-1$$ (4)

and then taking the geometric mean of *R* among all years for each route. Thus, *R* = 0 across the 17-year study period indicated a stable population, *R* > 0 indicated a growing population, and *R* < 0 indicated a declining population. We similarly used the above equation but took a geometric mean of R among all years and focal or paired routes, and among all years and routes to get overall focal area, paired area, and study area trends in relative abundance, respectively.

We used Markov Chain Monte Carlo and a Gibbs sampler in JAGS [35] using the jagsUI package [36] in program R [37] to obtain posterior distributions for all model parameters. We discarded at minimum the first 4,000 samples as burn-in, used a thinning rate of two, and saved at least 12,000 total samples from three chains for all models. We evaluated model convergence of chains using the Gelman-Rubin statistic ($\hat{R}$ < 1.1; [38]) and visual inspection of trace plots. We reported the geometric mean population growth rates and proportion of posterior samples above (for positive effects) or below (for negative effects) 0 as evidence that *R* or *β* were positive or negative.

## Results

### Abundance trends

**Fig 2:**
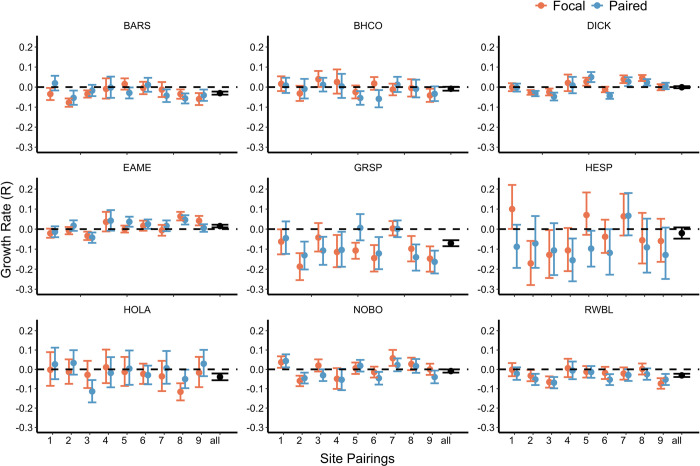
Relative abundance trends by species. Estimated trends in relative abundance (*R*) across all study years of barn swallow (BARS), brown–headed cowbird (BHCO), dickcissel (DICK), eastern meadowlark (EAME), grasshopper sparrow (GRSP), Henslow’s sparrow (HESP), horned lark (HOLA), northern bobwhite (NOBO), and red–winged blackbird (RWBL) at each site. Positive *R* values indicate a growing population whereas negative *R* values indicate a declining population. Error bars represent the 95% credible intervals for relative abundance trend estimates. Focal areas are depicted in orange and paired areas are depicted in blue. Trends across all areas are depicted in black.

Although estimated annual trends in species relative abundance were highly variable over time and across sites, species relative abundance trends were similar in virtually all cases among corresponding focal and paired areas (Table 2, Fig 2, S3–S11 Figs). Dickcissel relative abundance trends were positive (Pr(R > 0) = 0.893) in focal areas, but negative (Pr(*R* < 0) = 0.983) in paired areas, and Henslow’s sparrow relative abundance trends were stable (Pr(R > 0) = 0.519) in focal areas but negative (Pr(*R* < 0) = 0.997) in paired areas (Table 2). Across all sites during the 17-year study period, we found evidence of declines in barn swallow, brown-headed cowbird, grasshopper sparrow, Henslow’s sparrow, horned lark, northern bobwhite, and red-winged blackbird relative abundances (Pr(*R* < 0) = 0.919–1; Fig 2). We found evidence of increasing relative abundances of eastern meadowlarks (Pr(*R* > 0) = 1) across all sites during the study period (Fig 2).

**Table 2:** Estimated effects of site and landcover on abundances of grassland bird species across focal and paired areas within Missouri grasslands.

Species	Parameter	Lower 25%	Median	Upper 95%	*f *
Barn swallow	*focal *	-0.134	0.078	0.304	0.775
	*grass250 *	0.095	0.196	0.302	1
	*grass2500 *	0.418	0.908	1.419	1
	*R *	-0.038	-0.030	-0.022	1
	*R focal*	-0.043	-0.033	-0.022	1
	*R paired*	-0.038	-0.027	-0.016	1
Brown-headed cowbird	*focal *	-0.094	0.121	0.311	0.887
	*grass250 *	0.150	0.308	0.454	1
	*grass2500 *	-0.285	0.322	0.867	0.836
	*R *	-0.018	-0.008	0.002	0.954
	*R focal*	-0.017	-0.005	0.008	0.778
	*R paired*	-0.027	-0.013	0.002	0.956
Dickcissel	*focal *	-0.131	0.171	0.424	0.898
	*grass250 *	0.371	0.424	0.474	1
	*grass2500 *	-1.059	-0.781	-0.391	1
	*R *	-0.006	-0.001	0.005	0.624
	*R focal*	-0.002	0.004	0.012	0.893
	*R paired*	-0.014	-0.008	-0.001	0.983
Eastern meadowlark	*focal *	-0.094	0.152	0.395	0.903
	*grass250 *	0.682	0.756	0.830	1
	*grass2500 *	-0.996	-0.479	0.035	0.968
	*R *	0.009	0.015	0.022	1
	*R focal*	0.005	0.014	0.024	1
	*R paired*	0.008	0.017	0.026	1
Grasshopper sparrow	*focal *	-0.374	0.129	0.638	0.701
	*grass250 *	1.882	2.089	2.300	1
	*grass2500 *	3.238	4.365	5.503	1
	*R *	-0.085	-0.071	-0.055	1
	*R focal*	-0.093	-0.073	-0.053	1
	*R paired*	-0.088	-0.067	-0.045	1
Henslow’s Sparrow	*focal *	-0.575	0.767	1.762	0.897
	*grass250 *	2.053	2.542	3.022	1
	*grass2500 *	1.877	3.630	5.967	1
	*R *	-0.048	-0.020	0.008	0.919
	*R focal*	-0.035	-0.001	0.036	0.519
	*R paired*	-0.099	-0.060	-0.019	0.997
Horned lark	*focal *	-0.926	0.023	1.070	0.521
	*grass250 *	-3.244	-2.938	-2.627	1
	*grass2500 *	2.328	3.358	4.468	1
	*R *	-0.056	-0.039	-0.021	1
	*R focal*	-0.078	-0.053	-0.028	1
	*R paired*	-0.050	-0.027	-0.003	1
Northern bobwhite	*focal *	-0.093	0.088	0.269	0.861
	*grass250 *	0.139	0.247	0.352	1
	*grass2500 *	-0.993	-0.505	0.196	0.938
	*R *	-0.017	-0.009	0.000	0.979
	*R focal*	-0.015	-0.004	0.007	0.764
	*R paired*	-0.026	-0.015	-0.003	0.989
Red-winged blackbird	*focal *	-0.403	0.030	0.528	0.549
	*grass250 *	-0.827	-0.708	-0.584	1
	*grass2500 *	2.013	2.551	3.118	1
	*R *	-0.039	-0.031	-0.023	1
	*R focal*	-0.037	-0.026	-0.014	1
	*R paired*	-0.049	-0.037	-0.026	1

Median estimates, 95% credible intervals, and the proportion the posterior distribution greater than (for positive effects) or less than (for negative effects) 0 (*f*) for each variable from each species-specific model. Variables included whether a site was focal or paired (*focal*), percent grassland within the 250 m, local-level buffer (*grass250*), and percent grassland within the 2,500 m, landscape-level buffer (*grass2500*). Also shown are the species trends across all study years (2001–2017) and study sites (*R*), as well as species trends across all study years and all sites designated focal (*R focal*) or paired (*R paired*).

### Focal and paired areas

Barn Swallow, brown-headed cowbird, dickcissel, eastern meadowlark, grasshopper sparrow, Henslow’s sparrow, and northern bobwhite relative abundances were greater in focal than paired areas (Pr(*β*_1_ > 0) = 0.701–0.903; Table 2, S2 Fig). Horned lark and red-winged blackbird relative abundances did not differ between focal and paired areas (Pr(*β*_1_ > 0) = 0.521–0.548, Table 2, S2 Fig).

### Grassland cover

**Fig 3:**
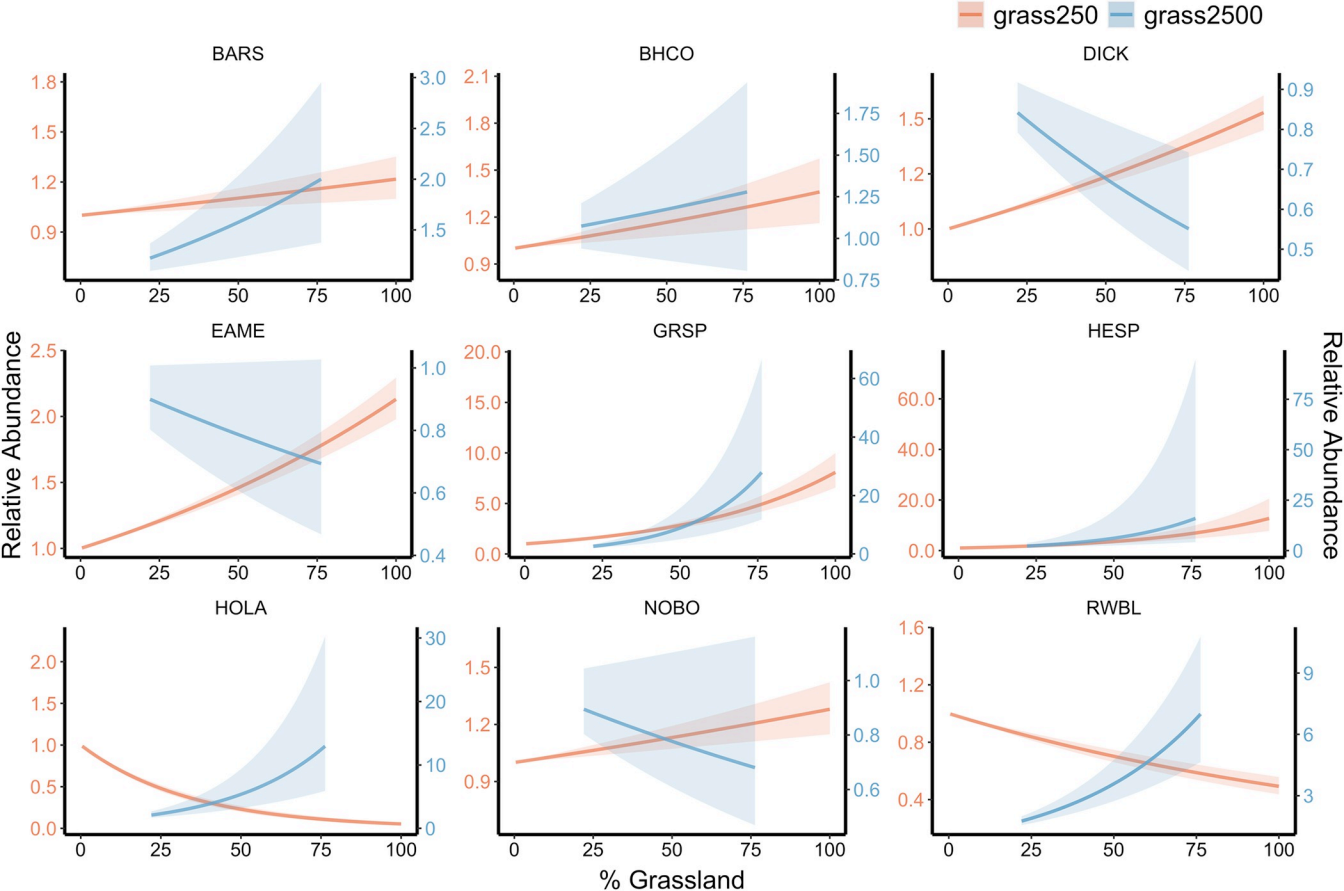
Local and landscape effect of grassland on relative abundance by species. Predicted relative abundance of barn swallow (BARS), brown–headed cowbird (BHCO), dickcissel (DICK), eastern meadowlark (EAME), grasshopper sparrow (GRSP), Henslow’s sparrow (HESP), horned lark (HOLA), northern bobwhite (NOBO), and red–winged blackbird (RWBL) in response to changes in grassland cover within the 250–m local–scale buffer (*grass250*, depicted in orange), and changes in grassland cover within the 2,500–m landscape–scale buffer (*grass2500*, depicted in blue) over the 17–year time period. Shaded areas represent 95% credible intervals.

At the local scale, horned lark and red-winged blackbird relative abundance decreased with increasing grassland cover (Pr(*β*_2_ < 0) = 1; Fig 3, S2 Fig). Relative abundance of all other species increased with increasing grassland cover at the local scale (Pr(*β*_2_ > 0) = 1; Fig 3, S2 Fig). At the landscape scale, dickcissel, eastern meadowlark, and northern bobwhite relative abundance decreased with increasing grassland cover (Pr(*β*_3_ < 0) = 0.938–1; Fig 3, S2 Fig). Barn Swallow, brown-headed cowbird, grasshopper sparrow, Henslow’s sparrow, horned lark, and red-winged blackbird relative abundance increased with increasing grassland cover at the landscape scale (Pr(*β*_3_ > 0) = 0.836–1; Fig 3, S2 Fig).

## Discussion

Long-term monitoring of avian populations is a crucial source of information for conservation planning, particularly for rapidly declining species guilds like grassland birds, which have undergone major population declines in North America [1, 39, 40]. North American Breeding Bird Survey data in Missouri since its initiation in 1966 indicated long-term declines in barn swallows, brown-headed cowbirds, dickcissels, eastern meadowlarks, grasshopper sparrows, horned larks, and northern bobwhites and declines during our 2001–2017 study period for barn swallows, brown-headed cowbirds, grasshopper sparrows, northern bobwhites, and red-winged blackbirds [27]. We similarly found evidence of declines in relative abundance across all sites for barn swallows, brown-headed cowbirds, grasshopper sparrows, Henslow’s sparrows, northern bobwhites, and red-winged blackbirds, though trends varied across the state. Our findings add to the body of evidence of declining populations of grassland bird species, including abundant species, throughout Missouri [28, 41] and the midwestern United States [1, 27].

The recent awakening to the large-scale losses of birds in North America and in particular grassland birds [1–3] may provide some insight into the variable success of local and landscape management for these species. Previous studies have demonstrated varied responses to habitat management across grassland birds [42–44]. We found evidence that while GC focal areas had higher relative abundances of grassland birds than paired sites (though the strength of this relationship varied among species and across sites), many of the long-term trends in relative abundance were similar between focal and paired sites (Fig 2, Table 2). Lack of detailed management practices and histories within focal areas over most of the study precluded examining the extent to which specific management practices affected species relative abundance and/or composition in focal areas. However, it’s likely that overall emphasis on grassland management in general on these areas allowed for higher numbers of our study species, though management did not appear to be sufficient to reverse declines in most species over our study period. Relative abundance trends were improved in focal compared to paired areas for dickcissels and Henslow’s sparrows. Both these species are grassland obligate and prefer dense grass cover [28, 29]; management on focal areas may have therefore improved these habitat conditions enough to reverse declines in abundance of these species. Despite the potential for grassland management to increase relative abundances, the temporal patterns in relative abundance across most focal and paired groups were very similar (Table 2, S3–S11 Figs). This observation suggests that population trends on these areas may actually be driven by forces at scales even beyond the landscape and instead relate to regional and global changes, thus limiting the effectiveness of local and landscape management.

There is increasing evidence that both local- and landscape-scale factors are major drivers of grassland bird abundance, and many conservation efforts attempt to implement multi-scale management goals [45–47]. We found that relative abundance of barn swallows, brown-headed cowbirds, dickcissels, eastern meadowlarks, grasshopper sparrows, Henslow’s sparrows, and northern bobwhites increased with increasing grassland cover at the local scale. This is consistent with the ecology of these species, as all use grassland habitat, and many are considered as grassland obligate [29]. Prior multi-scale research on drivers of grassland bird abundances similarly demonstrated the importance of local-scale habitat factors, including grassland vegetative structure, to grassland birds [48–50]. While horned larks and red-winged blackbirds utilize landscapes containing grasslands, horned larks prefer areas with short, sparse vegetation or plowed fields and red-winged blackbirds use a variety of upland and wetland habitats [51, 52]. Grassland may therefore not be as important to these species at the local scale as at other spatial scales.

Prior research has demonstrated the importance of grassland quantity at the landscape scale to grassland bird abundance, and landscape-scale declines in grassland quantity have been linked to population declines in grassland bird species [2, 53]. We did not detect a positive effect of grassland cover at the landscape scale on relative abundance of dickcissels, eastern meadowlarks, or northern bobwhites. However, many grassland birds are highly sensitive to grassland patch area, which can constrain the benefits of increasing grasslands overall for such species, when those grasslands are fragmented [54–56]. Much of Missouri’s remaining tallgrass prairie is highly fragmented [18, 19, 22]. A lack of management and the fragmentation of newly added habitat is likely why we did not detect a positive relationship between grassland cover at the landscape scale and relative abundance of dickcissels, eastern meadowlarks, or northern bobwhites. Future efforts to track grassland management (e.g., prevalence of high-quality and contiguous grassland) at a landscape scale would allow us to better understand how landscape-scale processes affect grassland bird relative abundance in Missouri.

A limitation of our study is our inability to account for imperfect detection in GC point count survey data. Unaccounted variability in detection probability of counted birds can potentially bias abundance and trend estimates from point count surveys [57, 58]. In response to such issues, many survey protocols such as distance sampling, double-observer sampling, time-of-detection sampling, and repeated counts have been designed to account for imperfect detection of individual birds [59–61]. Despite the development of such methods, many avian monitoring surveys continue to use methods that do not account for imperfect detection (e.g., North American Breeding Bird Survey [57] protocols) due to the need for simple protocols to maintain necessary volunteer observers. We believe that relative abundance indices and trends derived from data like the GC surveys provide valuable information in many contexts [62]. We believe the patterns emerging from even single visit models can help provide evidence of true abundance and trends, and guide future study designs. Including either distance or time-of-detection sampling in future GC surveys would allow for estimation of detection probability during statistical analysis and help researchers further assess if focal areas are meeting objectives of improving grassland bird abundance.

The use of roadside point count surveys may also cause limitations. Surveys during our study were often conducted adjacent to, rather than within large tracts of grassland, as roads did not exist within these contiguous grasslands. Areas surrounding roads can be less reflective of grassland management, potentially resulting in fewer observations of grassland obligate species [63, 64] and decreasing our ability to detect positive relationships between abundance and grassland cover. More direct and targeted monitoring may help researchers better understand relationships between habitat and abundance of our study species. However, we believe long-term roadside point count datasets like the one used in our study can provide crucial information about the population trends of many species of conservation concern. Our study provides one of the only long-term assessments of abundance trends in several bird species of conservation concern in Missouri, as well as the only analysis assessing how trends in abundance of these species may be affected by a long-term conservation effort. Thus, we believe our study provides valuable information for conservation practitioners in this region.

## Conclusions

In response to declines in grassland bird abundance, many agency- and nonprofit organization-sponsored conservation programs have been implemented to help secure wildlife habitat on private and public lands. However, few research efforts have examined the effectiveness of these programs in improving conservation outcomes, resulting in an incomplete understanding of the benefits to grassland bird abundance. We analyzed long-term point count data to assess the effectiveness of a regional habitat management partnership in improving relative abundance and population trends of nine grassland bird species in Missouri. We found evidence of increased relative abundance of several grassland bird species in focal than paired areas, possibly resulting from efforts to increase availability of grassland habitat at landscape scales and increase quality at local scales with management. However, further efforts to decrease landscape fragmentation and improve grassland habitat quality may be needed to fully realize grassland bird conservation goals. Future efforts to monitor grassland songbird trends on focal grasslands and paired areas in Missouri would be improved by conducting point counts off-road within focal areas, incorporating methods to account for imperfect detection, and vegetation monitoring over time at specific survey points and across the landscape. Such sampling adaptations could improve our understanding of scale-specific land cover and focal areas affecting grassland bird abundance, which are critical steps in reversing declines of this imperiled species guild.

## Supporting information

S1 Appendix Modifications to improve model convergence. (PDF)

S1 Fig Grassland cover characteristics of study sites at local and landscape scales. (PDF)

S2 Fig Violin plots of covariate posterior means by species. (PDF)

S3 Fig Barn swallow estimated relative abundance by site. (PDF)

S4 Fig Brown-headed cowbird estimated relative abundance by site. (PDF)

S5 Fig Dickcissel estimated relative abundance by site. (PDF)

S6 Fig Eastern meadowlark estimated relative abundance by site. (PDF)

S7 Fig Grasshopper sparrow estimated relative abundance by site. (PDF)

S8 Fig Henslow’s Sparrow estimated relative abundance by site. (PDF)

S9 Fig Horned lark estimated relative abundance by site. (PDF)

S10 Fig Northern bobwhite estimated relative abundance by site. (PDF)

S11 Fig Red-winged blackbird estimated relative abundance by site. (PDF)
