# Supplementary material for: Long-term trends in grassland bird relative abundance on focal grassland landscapes in Missouri
Source: PLoS One. 2023 Mar 9;18(3):e0281965. doi: 10.1371/journal.pone.0281965 (PMC9997899; doi:10.1371/journal.pone.0281965)
Supplement: S1 Appendix — (PDF) [file pone.0281965.s001.pdf]

S1 Appendix. Modifications to improve model convergence.

We further altered the Dickcissel and Red-winged Blackbird models to improve convergence. Dickcissel count data did not contain a large proportion (<25%) of non-detections; thus, we assumed data for this species was Poisson distributed such that

$$y_{k,a,t} \sim Poisson(\lambda_{k,a,t}). \tag{1}$$

where $y_{k,a,t}$, comprised count data from surveys at route *k* and point *a* in year *t* and $\lambda_{k,a,t}$ was the expectation of count data $y_{k,a,t}$. The rest of the model was parameterized the same as in other species.

Red-winged Blackbird count data contained several large (>100) counts, resulting in overdispersion. To account for overdispersion, we specified data as coming from a negative binomial distribution such that

$$y_{k,a,t} \sim NegBin(p_{k,a,t}, r), \tag{2}$$

where $y_{k,a,t}$, comprised count data from surveys at route *k* and point *a* in year *t*, *r* was the overdispersion parameter, $p_{k,a,t}$ the probability of success, and mean abundance $\mu_{k,\mathrm{a},t} = r(1 - p_{k,a,t}) / p_{k,a,t}$. This parameterization allowed for the variance in abundance to be larger than the mean ($\mu_{k,a,t}$). We constructed an ecological process model similarly to other species:

$$\log(m_{k,a,t}) = \beta_{1x_k^{managed}} + \beta_{2x_{k,a,t}^{grass250}} + \beta_{3x_{k,t}^{grass2500}} + \varepsilon_{k,t}. \tag{3}$$

We assumed the same priors for all parameters as in our other models.
