# Supplementary material for: Long-term trends in grassland bird relative abundance on focal grassland landscapes in Missouri
Source: PLoS One. 2023 Mar 9;18(3):e0281965. doi: 10.1371/journal.pone.0281965 (PMC9997899; doi:10.1371/journal.pone.0281965)

S1 Fig. Grassland cover characteristics of study sites at local and landscape scales. Boxplots showing the distribution of grassland cover at the local (250-m buffer) and landscape (2,500-m buffer) scales among all years for each study site. Focal areas are depicted in orange and paired areas are depicted in blue.

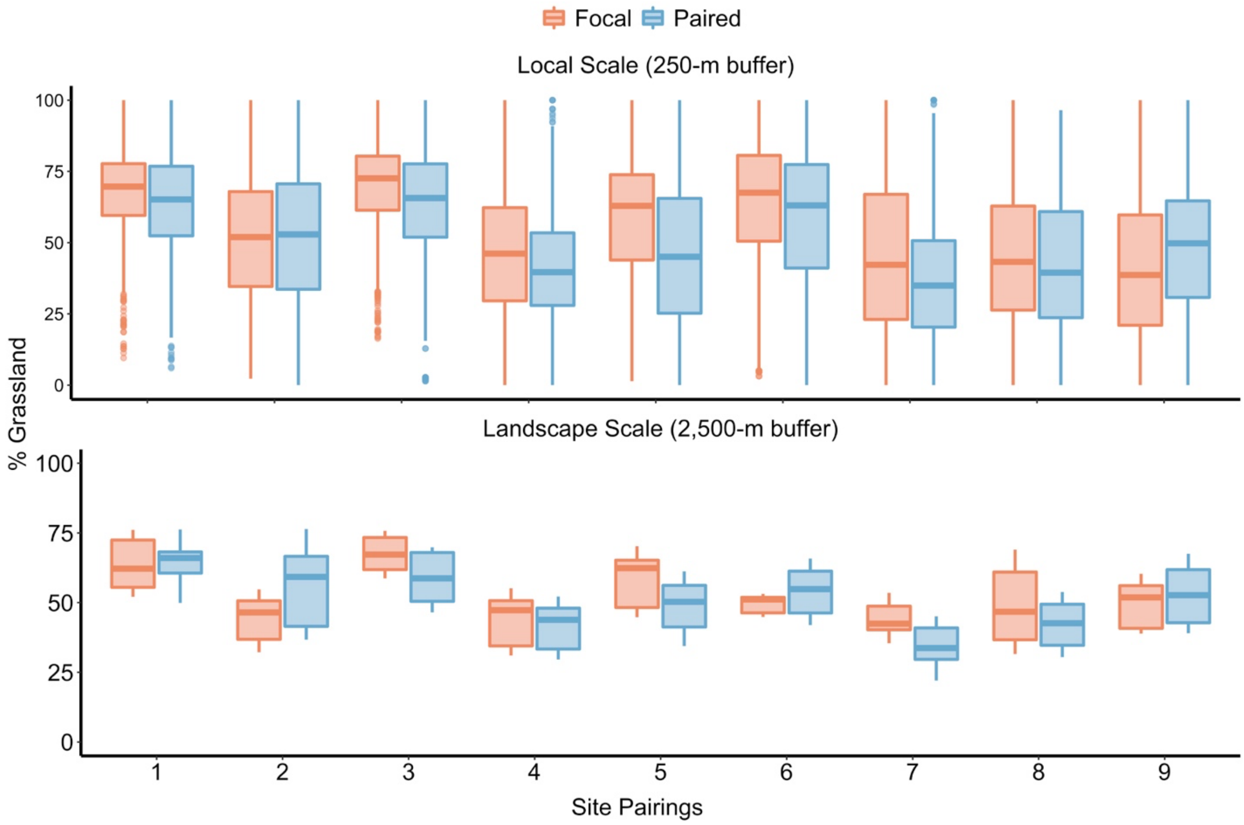

Supplement: S1 Fig — (PDF) [file pone.0281965.s002.pdf]
