# Supplementary material for: Long-term trends in grassland bird relative abundance on focal grassland landscapes in Missouri
Source: PLoS One. 2023 Mar 9;18(3):e0281965. doi: 10.1371/journal.pone.0281965 (PMC9997899; doi:10.1371/journal.pone.0281965)

S2 Fig. Violin plots of covariate posterior means by species. Violin plots showing posterior means for the effects of whether a site was focal or paired (*focal*), grassland cover within the 250 m, local-scale buffer (*grass250*), and grassland cover within the 2,500-m landscape-scale buffer (*grass2500*) throughout the full 17-year period on relative abundance of barn swallow (BARS), brown-headed cowbird (BHCO), dickcissel (DICK), eastern meadowlark (EAME), grasshopper sparrow (GRSP), Henslow's Sparrow (HESP), horned lark (HOLA), northern bobwhite (NOBO), and red-winged blackbird (RWBL). Posterior means indicate the strength of the corresponding covariate effect. Error bars represent 95% credible intervals.

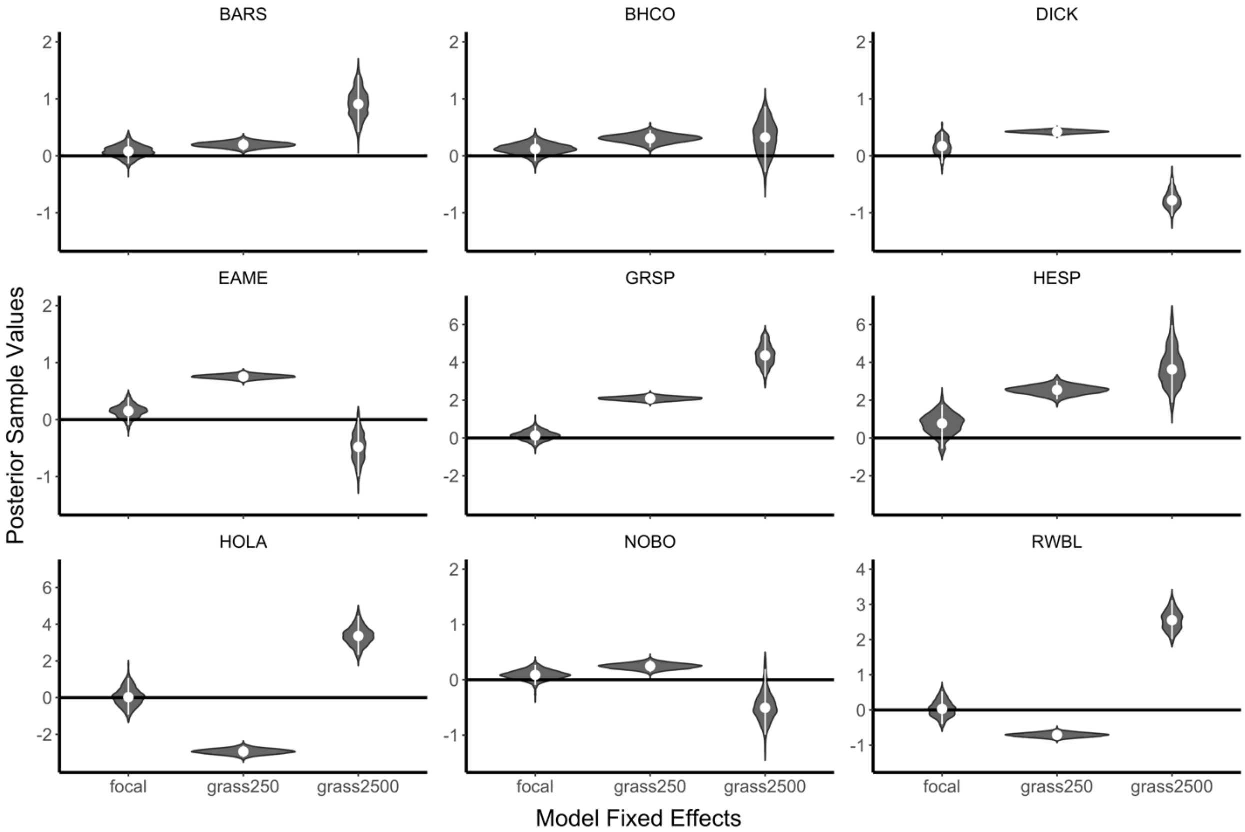

Supplement: S2 Fig — (PDF) [file pone.0281965.s003.pdf]
