# Supplementary material for: Long-term trends in grassland bird relative abundance on focal grassland landscapes in Missouri
Source: PLoS One. 2023 Mar 9;18(3):e0281965. doi: 10.1371/journal.pone.0281965 (PMC9997899; doi:10.1371/journal.pone.0281965)

S10 Fig. **Northern bobwhite estimated abundance by site.** Estimated abundance of Northern bobwhites (NOBO) for each pairing of sites 2001-2017, with shaded areas representing 95% credible intervals. Focal areas are depicted in orange and paired areas depicted in blue.

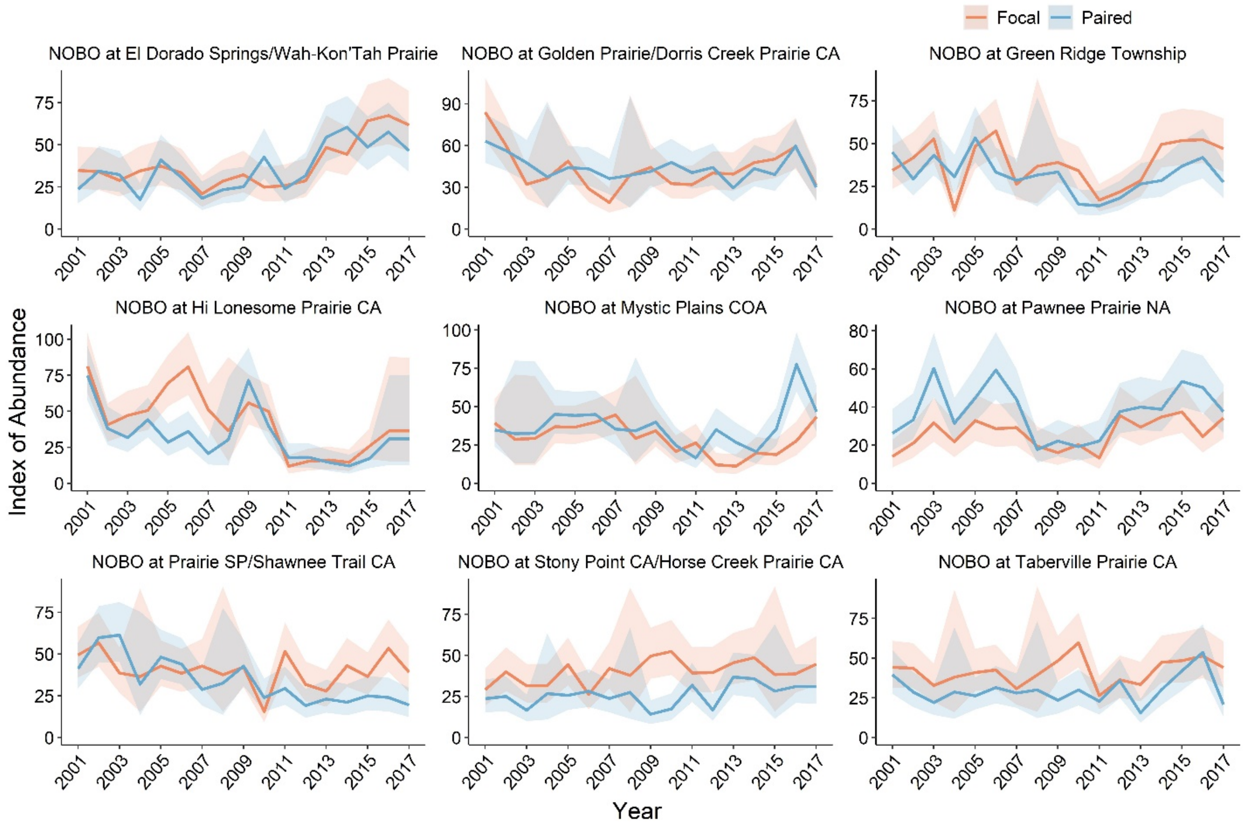

Supplement: S10 Fig — (PDF) [file pone.0281965.s011.pdf]
